# p16 is superior to Stathmin-1 and HSP27 in identifying cervical dysplasia

**DOI:** 10.1186/s13000-021-01144-w

**Published:** 2021-09-20

**Authors:** Sofia Liou, Neshat Nilforoushan, Yuna Kang, Neda A. Moatamed

**Affiliations:** Department of Pathology & Laboratory Medicine, David Geffen School of Medicine at UCLA, 10833 Le Conte Avenue, BOX 951732, 13-145 CHS, Los Angeles, CA 90095-1732 USA

**Keywords:** Cervix, Dysplasia, Adenocarcinoma, Stathmin-1, HSP27, P16

## Abstract

**Background:**

The aim of this study was to determine how Stathmin-1 and Heat Shock Protein 27 (HSP27) can be used as adjunctive biomarkers to differentiate high-grade dysplasia from benign/reactive lesions in cervical tissues. In addition, we aimed to see if any of these markers can differentiate endometrial from endocervical adenocarcinomas.

**Methods:**

Fifty cases including benign cervical tissue, low-grade squamous intraepithelial lesion (LSIL), high-grade squamous intraepithelial lesion (HSIL), adenocarcinoma in situ of the endocervix, invasive endocervical adenocarcinoma, and endometrial adenocarcinoma were selected. Stathmin-1 and HSP27 immunohistochemistry (IHC) were performed for each case and the results were compared to the previously available p16 IHC stains.

**Results:**

p16 stained positively in 100% of HSIL, endocervical adenocarcinoma in situ, and invasive endocervical cases. Stathmin-1 stained positively in 43% of HSIL and 90% of endocervical adenocarcinoma in situ and all invasive endocervical cases. Stathmin-1 and p16 were negative in all benign cervical samples. Stathmin-1, HSP27, and p16 stained 100% of LSIL cases. HSP27 stained indiscriminately, including 100% of benign cervical tissue. 87% of the endometrial adenocarcinomas stained positively for p16, Stathmin-1, and HSP27.

**Conclusion:**

p16 remains superior to both Stathmin-1 and HSP27 in differentiating dysplasia from benign, reactive changes of the cervix.

**Supplementary Information:**

The online version contains supplementary material available at 10.1186/s13000-021-01144-w.

## Background

The diagnostic distinction between cervical dysplasia and benign lesions can be challenging when the evaluation is limited to the assessment of the hematoxylin and eosin (H&E) stained slides. Ancillary biomarkers can aid in making accurate diagnoses [1]. The most important ancillary studies when assessing for cervical dysplasia are p16 and Ki67 immunohistochemistry (IHC) stains, as well as in situ hybridization for human papillomavirus (HPV) RNA [2–4]. Other IHC stains which have been proposed for the use in distinguishing benign conditions of the cervix from dysplastic lesions include cyclin D1, P53, and ProEx C in various organs [5–7].

Oncoprotein 18, also known as Stathmin-1, is an important cell-cycle protein [8]. It is expressed in a variety of malignancies and can predict the aggressiveness of tumors [9, 10]. Prior studies have also proposed Stathmin-1 and Heat Shock Protein 27 (HSP27) as adjunct biomarkers which can help differentiate high-grade dysplasia from low-grade dysplasia and benign/reactive cervical tissue [1, 11–13]. Stathmin-1 has been reported to interact with heat shock protein 27 (HSP27), which is an inhibitor of cyclin-dependent kinases and can promote the proliferation of tumor cells [14]. Overexpression of HSP27 has been reported in a number of malignancies [15–21] including squamous cell carcinoma of the cervix [11]. High-grade squamous intraepithelial lesion (HSIL), low-grade squamous intraepithelial lesion (LSIL), endocervical adenocarcinoma in situ (AIS) and usual type invasive endocervical adenocarcinoma are human papillomavirus (HPV)-driven lesions; therefore, HPV RNA in-situ hybridization is an important ancillary test to support these diagnoses [22].

In this study, we investigated Stathmin-1and HSP27in comparison to p16 expression in benign cervix, LSIL, HSIL, AIS, usual type invasive adenocarcinoma of the cervix, and endometrial endometrioid adenocarcinoma. We have compared the test performances of each antibody to see whether Stathmin-1 and HSP27 can serve as additional markers for the diagnosis of cervical dysplasia.

## Materials and methods

This study was reviewed and approved by the Institutional Review Board at David Geffen.

School of Medicine at UCLA (IRB# 17–001254). This retrospective study was carried out by obtaining data through a computer search of our departmental database (Epic Beaker, Atlanta, Georgia). Search criteria included cervical, endocervical, and endometrial biopsies which had p16 IHC performed for the period of April 11, 2016 to March 11, 2019. Also, the cases were further filtered by availability of unstained duplicate glass slides for the additional IHCs. Stathmin-1 (clone: SP49) rabbit monoclonal antibody (cellmarque.com), HSP27 (clone: G3.1) mouse monoclonal antibody (sigmaaldrich.com) [23], and p16 (clone: E6H4) mouse monoclonal antibody (roche.com) [24] were used. IHC procedures were performed according to the published protocols in the manufacturers’ product insert with appropriate positive and negative controls. p16 is an accepted marker for HSIL or usual type adenocarcinoma of endocervix when block/diffuse staining of nuclei is present, with or without cytoplasmic reactivity [1]. Stathmin-1 reactivity is mainly cytoplasmic and is present in the basal cell layer of normal cervical squamous mucosa, also considered as an internal positive control based on the published criteria for interpretation of the IHC. Stathmin-1 positivity in dysplasia involves at least two-third of the squamous epithelial thickness [1]. For HSP27, the results were considered positive when more than 5% of the cells showed expression of the protein, using the published criteria for the IHC interpretation [23]. p16 was considered positive for HPV related high grade squamous dysplasia or adenocarcinoma when block/diffuse staining of nuclei, with or without cytoplasmic reactivity, was noted [1].

### Study design

Stathmin-1, HSP27, and p16 results were tabulated into six histopathology diagnostic categories: Benign, LSIL, HSIL, endocervical adenocarcinoma-in-situ (AIS), usual type invasive endocervical adenocarcinoma (UIEACa), and endometrial endometrioid adenocarcinoma (EACaET). Of which, LSIL, HSIL, AIS, and UIEACa were treated as HPV-associated lesions in this study. The stains were analyzed primarily in the context of HPV surrogate markers, where the benign lesions and the endometrial cancers were included as controls (Supplementary Table 1). All positive IHC reactions in the HPV-associated lesions were considered as true positive (TP), while their negativity was deemed as false negative (FN). The positive stains in the “benign” and “EACaET” categories were treated as false positive (FP) and true negative (TN) when the reactions were absent (Supplementary Table 1). Sensitivity, specificity, positive predictive value (PPV), negative predictive value (PPV), and diagnostic accuracy (DA) were determined for Stathmin-1, HSP27, and p16 [25]. “Benign” patients were added to each diagnostic category for the test parameter analyses. Binary representations of the results were also recoded as 0 (negative) or 1 (positive) for Student’s T-test analyses (Supplementary Table 1). Student’s t-tests were carried out excluding the EACaET lesions from the analyses. A one-tailed *p*-value of 0.05 or less was considered a significant statistical difference between the two compared sets of data.

## Results

**Fig. 1:**
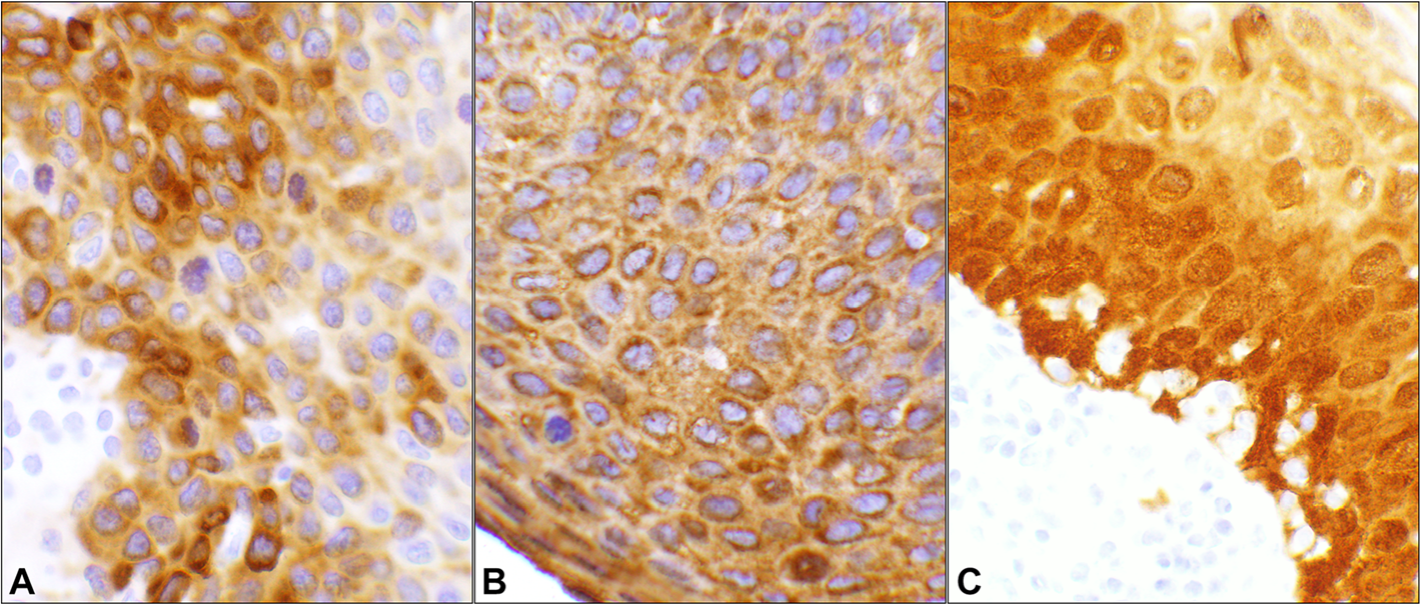
Immunohistochemical (IHC) reactivity pattern of the three markers. The three photomicrographs are obtained from a case of High grade squamous intraepithelial lesion (HSIL) (Case# 22, Supplemental Table 1). **A** Stathmin-1 shows the perinuclear cytoplasmic staining involving more than two third of the epithelial thickness. **B** HSP27 immunohistochemistry shows cytoplasmic staining of the dysplastic squamous cells covering the full thickness of the epithelium. **C** p16 positive immunostain of the entire epithelium which involves both nucleus and cytoplasm, but more intensely depicting the nuclei. (Objective 40x)

There were 50 cases in this series of which 35 patients had HPV-associated lesions (Supplementary Table 1). While the p16 reaction was predominantly nuclear, Stathmin-1 and HSP27 staining’s were mainly cytoplasmic (Fig. 1). As HPV surrogate markers, Stathmin-1 and HSP27 had a sensitivity of 71 and 94% while their specificity was 53 and 7% respectively for the combined diagnostic categories. Other test measures were listed in the table where the two parameters were 100 and 53% for p16, respectively (Table 1). Overall, diagnostic accuracies for Stathmin-1, HSP27, and p16 were 66, 68, and 86%, respectively. Using Student’s t-test, Stathmin-1 and HSP27’s *p*-values were 0.05 or less as compared to each other, p16, and histopathology diagnosis while the p16 analysis yielded a non-significant value of “1” when compared to the histopathology diagnoses (Table 1).

**Table 1:**
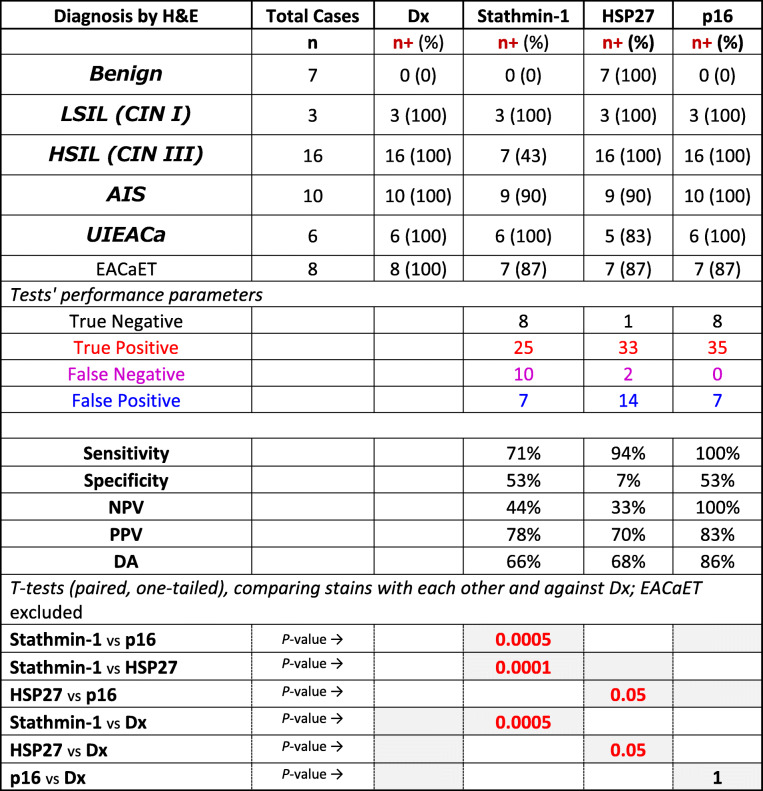
Test performance and T-test analyses of the stains in the HPV surrogate context

*H&E* Hematoxylin and eosin, *Dx* Histopathology diagnosis, n+ number of positive tests, *LSIL* Low grade squamous intraepithelial lesion, *HSIL* High grade squamous intraepithelial lesion, *CIN* Cervical intraepithelial neoplasia, *AIS* Adenocarcinoma in situ, endocervix, *UIEACA* Invasive endocervical adenocarcinoma, usual type, *EACaET* Endometrial adenocarcinoma, endometrioid type, *NPV* Negative predictive value, *PPV* Positive predictive value, *DA* Diagnostic accuracy, *vs* Versus; Red *P*-value, significant difference

### Benign

**Fig. 2:**
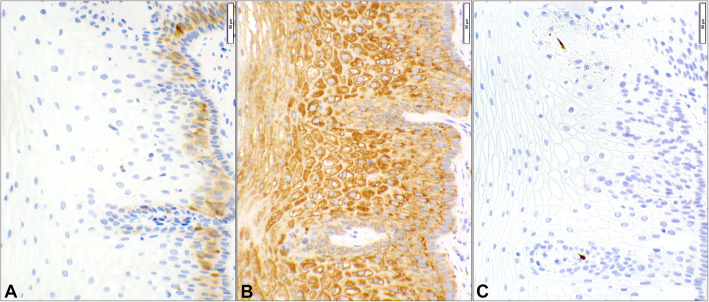
HSP27 false positivity in benign squamous epithelium. The three photomicrographs are obtained from a case of benign ectocervical tissue (Case #3, Supplemental Table 1). **A** Stathmin-1 shows weak positivity only in the basal and parabasal layers which is considered as negative. **B** HSP27 shows cytoplasmic staining of the full thickness of benign squamous epithelium. **C** p16 is completely negative in the squamous mucosa. (Objective 20x)

There were seven cases in this category with benign lesions (cases 1–7, Supplementary Table 1). The patients’ ages ranged from 28 to 87 years. Four of the 7 cases had a history of positive high-risk HPV results and the remaining three were negative. Histologically, four cases showed atrophic squamous epithelium and three had reactive/metaplastic changes. p16 and Stathmin-1 were negative in all these cases. HSP27 was positive in all 7 patients which were classified as *false positives* (Supplementary Table 1). These benign cases had diagnoses of atrophy, metaplasia, and other reactive changes. HSP27 was focally or weakly positive in three and diffusely positive in the rest of the cases (Supplementary Table 1). An example of a false positive HSP27 stain with true negative Stathmin-1 and p16 stains is shown in Fig. 2.

### Low grade squamous intraepithelial lesion (LSIL)

There were three patients in this category. The patients were 32 to 62 years of age. All cases had a history of positive high-risk HPV types other than 16 and 18. Stathmin-1, HSP27, and p16 IHCs were positive in all three cases. The staining patterns, for HSP27 and P16, were diffuse but the reaction was focal for Stathmin-1. All positive reactions were classified as *true positive* in this diagnostic category (Supplementary Table 1). All stains had a sensitivity and specificity of 100%, except for HSP27 which had a 0% specificity. Similarly, the diagnostic accuracy was 100% for Stathmin-1 and p16, while HSP27’s value was 30% (Table 2).

**Table 2:**
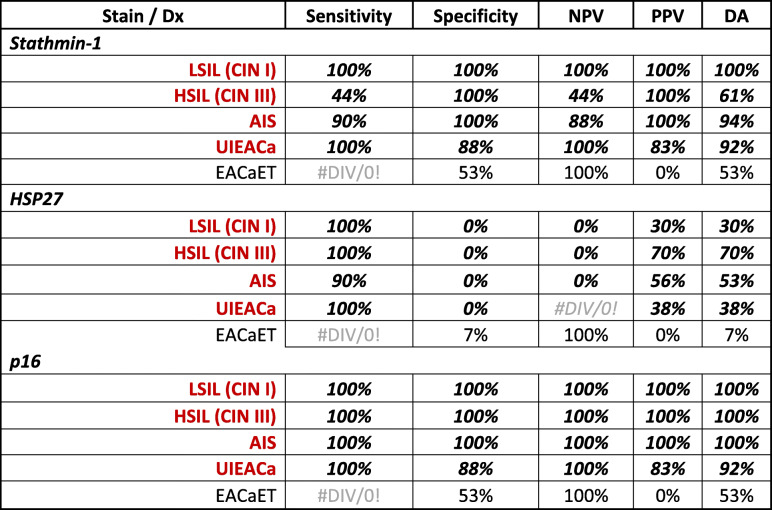
Test performance of the three stains for each histopathology diagnostic category

*Dx* histopathology diagnosis, Red, HPV related lesions, *LSIL* low grade squamous intraepithelial lesion, *HSIL* High grade squamous intraepithelial lesion, *CIN* Cervical intraepithelial neoplasia, *AIS* Adenocarcinoma in situ, endocervix, *UIEACA* Invasive endocervical adenocarcinoma, usual type, *EACaET* Endometrial adenocarcinoma, endometrioid type, *NPV* Negative predictive value, *PPV* Positive predictive value, *DA* Diagnostic accuracy

### High grade squamous intraepithelial lesion (HSIL)

**Fig. 3:**
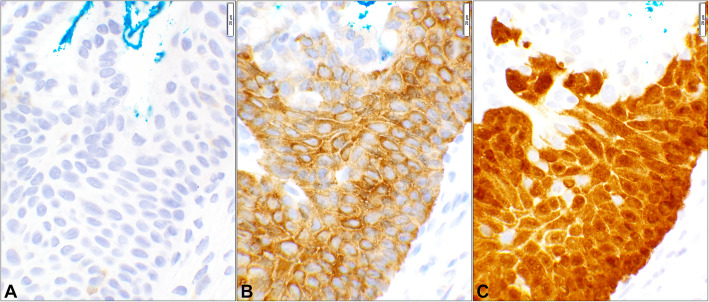
Stathmin-1 false negative in high grade squamous intraepithelial lesion. The three photomicrographs are obtained from a case of high grade squamous intraepithelial lesion (Case# 13, Supplemental Table 1). **A** Stathmin-1 shows negative staining of the dysplastic squamous epithelium. **B** HSP27 shows cytoplasmic staining of the dysplastic squamous cells. **C** p16 positive immunostain shows diffuse nuclear and cytoplasmic staining of the high grade squamous intraepithelial lesion/cervical intraepithelial lesion grade III. (Objective 40x)

There were sixteen subjects in this category. The patients’ ages ranged from 24 to 55 years. Twelve cases had a history of positive high-risk HPV. Among the remaining 4, one had a negative HPV result, and three cases did not have their results in our system. Stathmin-1 IHC stains were positive in seven cases which were classified as *true positive* and the remaining 9 subjects were listed as *false negative*. An example of a false negative Stathmin-1 stain with true positive p16 and HSP27 is shown in Fig. 3. HSP27 and p16 IHC stains were positive in all 16 cases which were classified as *true positive* (Supplementary Table 1). The sensitivities were 44, 100, and 100% for Stathmin-1, HSP27, and p16, while the specificities were 100%, for Stathmin-1 and p16, it was 0% for HSP27. The diagnostic accuracies were 61, 70, and 100% for Stathmin-1, HSP27, and p16, respectively. All the test performance parameters are listed in Table 2.

### Endocervical adenocarcinoma-in-situ (AIS)

There were ten cases in this category. The patients’ ages ranged from 30 to 47 years. Among these patients, the history of HPV positivity was present in 7 with type-16, one with type-18, and one with other high-risk types. One did not have her HPV results in our system. All three stains were positive in this diagnostic category except for Stathmin-1 in one case and HSP27 in another one which all were classified as *true positive* except for these two subjects which were listed as *false negative* (Supplementary Table 1). The sensitivities were 90, 90, and 100% while the specificities were 100, 0, and 100% for Stathmin-1, HSP27, and p16, respectively. All parameters including the diagnostic accuracies are listed in Table 2.

### Invasive endocervical adenocarcinoma, usual type (UIEACa)

**Fig. 4:**
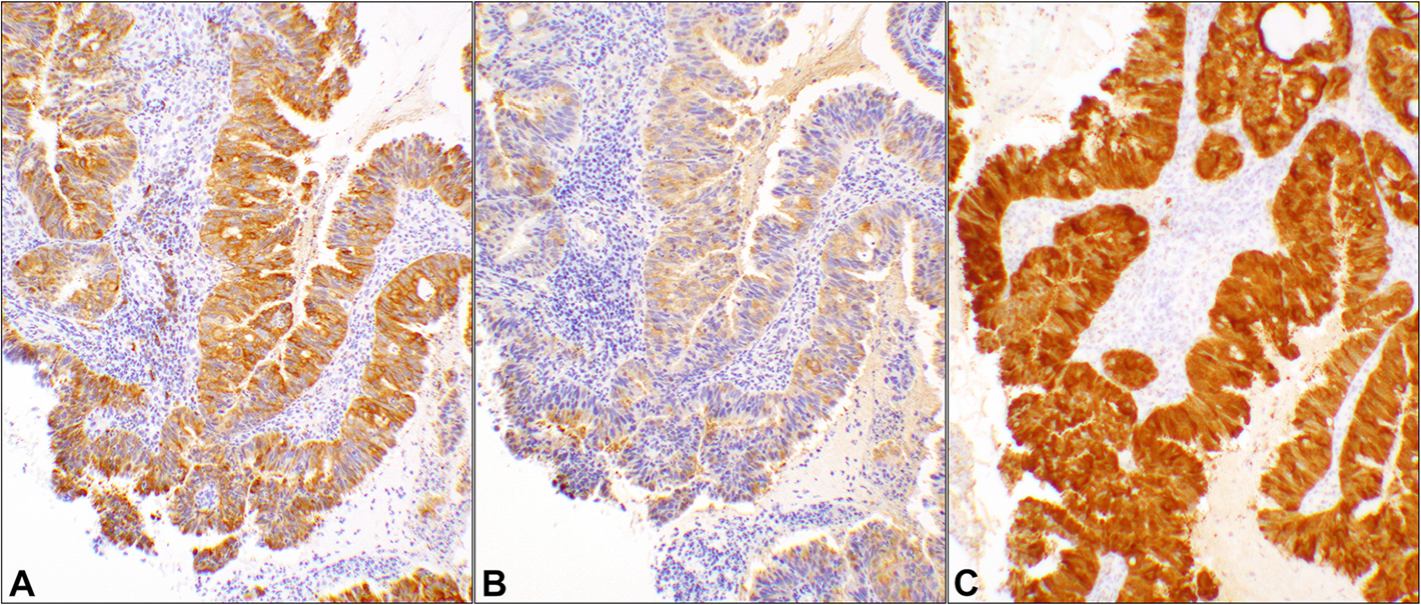
Positive IHC reactivity in invasive endocervical adenocarcinoma, usual type. The three photomicrographs are obtained from a case of invasive adenocarcinoma of the endocervix (Case #41, Supplemental Table 1). **A** Stathmin-1, **B** HSP27, and **C** p16 positive immunostains in invasive endocervical adenocarcinoma. (Objective 10x)

There were six patients in this category. The cases were 37 to 48 years old. HPV status was available only in two cases, one had type-16 and the other had type-16 plus other high-risk types. Examples of the IHC reactivities of the 3 stains are displayed in Fig. 4. All three stains were classified as *true positive* in this category except for one where HSP27 was recorded as *false negative* (Supplementary Table 1). In this category, the sensitivities were 100% for all three stains. However, the specificity was 88% for Stathmin-1 and p16 but 0% for HSP27. The five parameters for the stains are listed in Table 2.

### Endometrial endometrioid type adenocarcinoma (EACaET)

There were eight subjects in this category. The patients’ ages ranged from 58 to 81 years.

The type of endometrial adenocarcinoma was endometrioid with FIGO grade I in 4 cases, FIGO grade II in 2 cases, and FIGO grade III in 2 cases. Stathmin-1, HSP27, and p16 IHC reactions were positive in all cases except for 3, one with Stathmin-1, one with HSP27, and one with p16. It is noteworthy that the p16 positive staining was patchy and not as diffuse as in UIEACa. All positive cases were classified as *false positives* and the three (one for each stain) as *true negatives* in the context of HPV surrogate markers (Supplementary Table 1).

## Discussion

At the outset, this study confirms the superiority of p16 to Stathmin-1 and HSP27 in distinguishing HPV-related preneoplastic and neoplastic lesions of the cervix based on the test performance analyses. Not only, p16 offers a higher diagnostic accuracy in every HPV-related diagnostic categories (Table 2), it has also a far better DA collectively (86%) than the other two (66% & 68%) as shown in Table 1. Additionally, Student’s t-test has reaffirmed the significant differences between p16 and the other two markers (Table 1). Most substantial finding is close correlation of the histopathology diagnoses with p16 (*p*-value = 1) which is opposite (*p*-value ≤0.05) of the other two markers (Table 1). The main issue with Stathmin-1 is too many false negatives (Fig. 3) while HSP27 suffers from too many false positives (Fig. 2), resulting in a highly variable test performance.

Cervical biopsies are commonly performed for the diagnosis of squamous intraepithelial lesions and cervical cancers. Although in most of the cases the diagnosis of such lesions is feasible by morphology alone, there are situations where biomarkers are needed for distinction between benign and dysplastic entities. p16 IHC has been the most reliable surrogate marker in high-grade dysplasia and is broadly used along with Ki67 to differentiate benign mucosa and LSIL from HSIL; however, it can stain some benign or non-HPV related neoplasia. Previous studies have shown that Stathmin-1 and p27, two regulators of the cell cycle, can also be used to identify high-grade dysplasia [1]. Tozawa-Ono et al. have demonstrated that the combination of HSP27 and p16 will improve the sensitivity and specificity of identification of cervical dysplasia and cervical squamous cell carcinomas [13]. In view of the high number of false positive HSP27 stains in this study, its combination with p16 may lower the specificity of the identification.

Howitt et al. showed that Stathmin-1 IHC is a good marker for HSIL and invasive squamous cell carcinoma and can distinguish LSIL from HSIL [1]. These findings are contrary to ours. Although our series is a small one, it cannot substantiate Howitt, et al.’s study conclusion. When comparing these 3 IHC stains in endocervical versus endometrial adenocarcinoma, none could distinguish the two lesions. Other studies have shown that HPV in situ hybridization is very useful in the diagnostic evaluation of adenocarcinomas of the endocervix versus endometrium, which is recommended in the tumors of uncertain origin [26, 27]. Overall, our study showed that p16 IHC is superior to both Stathmin-1 and HSP27 IHC in differentiating the HPV-related preneoplastic from benign and reactive lesions. Among the selected subjects for this study, unfortunately we did no encounter cases with CIN II which might have added a dimension to this work. However, it does not seem to have impacted the conclusions, since Stathmin-1 and HSP27 have not emerged as superior substitutions for p16. In daily practice, it can be difficult to differentiate reactive changes from LSIL, or LSIL from CIN II, and HSIL/CIN II-III from immature metaplasia. Regrettably, stathmin-1 and HSP27 have not provided such a distinction, therefore, p16 remains as the IHC stain of choice for the time being.

## Conclusion

Until newer markers are introduced, p16 IHC remains as the HPV-surrogate marker of choice. Usage of Stathmin-1 and HSP27 IHC cannot compete with p16 in this regard and would not add any benefit in this diagnostic endeavor.

## Supplementary Information

**Additional file 1: Supplementary Table 1.** Summary of the immunohistochemistry stains’ results and their performance in each histopathology diagnostic category.

## Data Availability

All relevant data are within the paper and its Supporting Information files. The original Excel sheet and the statistical analyses results can be obtained from Harvard DataVerse: 10.7910/DVN/PRBFN9.

## Abbreviations

HSIL: High-grade squamous intraepithelial lesion
LSIL: Low-grade squamous intraepithelial lesion
AIS: Adenocarcinoma in situ
HPV: Human papillomavirus
IHC: Immunohistochemistry
H&E: Hematoxylin and eosin
CIN: Cervical intraepithelial neoplasia
UIEACa: Invasive endocervical adenocarcinoma, usual type
NPV: Negative predictive value
PPV: Positive predictive value
DA: Diagnostic accuracy
EACaET: Endometrial adenocarcinoma, endometrioid type
HSP27: Heat shock protein 27
