# Supplementary material for: p16 is superior to Stathmin-1 and HSP27 in identifying cervical dysplasia
Source: Diagn Pathol. 2021 Sep 20;16:85. doi: 10.1186/s13000-021-01144-w (PMC8451080; doi:10.1186/s13000-021-01144-w)
Supplement: Supplementary file 1 — Additional file 1: Supplementary Table 1. Summary of the immunohistochemistry stains’ results and their performance in each histopathology diagnostic category. [file 13000_2021_1144_MOESM1_ESM.pdf]

Supplementary Table 1. Summary of the immunohistochemistry stains' results and their performance in each histopathology diagnostic category

No	Age	HPV	*H&E Histopathology*		*Stathmin-1*			*HSP27*			*p16*		
			Abbreviated Dx	*Binary*	*Results*	*Perform*	*Binary*	*Results*	*Perform*	*Binary*	*Results*	*Perform*	*Binary*
			Benign, n = 7										
1	62	N	Benign	0	Negative	TN	0	Positive, Focal	FP	1	Negative	TN	0
2	51	HR	Benign	0	Negative	TN	0	Positive, Focal	FP	1	Negative	TN	0
3	28	N	Benign	0	Negative	TN	0	Positive	FP	1	Negative	TN	0
4	47	HR	Benign	0	Negative	TN	0	Positive, Weak	FP	1	Negative	TN	0
5	47	N	Benign	0	Negative	TN	0	Positive	FP	1	Negative	TN	0
6	35	HR	Benign	0	Negative	TN	0	Positive	FP	1	Negative	TN	0
7	87	HR	Benign	0	Negative	TN	0	Positive	FP	1	Negative	TN	0
			LSIL (CIN I), n = 3										
8	32	HR	LSIL (CIN I)	1	Positive, Focal	TP	1	Positive	TP	1	Positive	TP	1
9	62	HR	LSIL (CIN I)	1	Positive, Focal	TP	1	Positive	TP	1	Positive	TP	1
10	53	HR	LSIL (CIN I)	1	Positive, Focal	TP	1	Positive	TP	1	Positive	TP	1
			HSIL (CIN III), n = 16										
11	55	HR	HSIL (CIN III)	1	Negative	FN	0	Positive	TP	1	Positive	TP	1
12	34	N	HSIL (CIN III)	1	Positive, Focal	TP	1	Positive	TP	1	Positive	TP	1
13	43	HR	HSIL (CIN III)	1	Negative	FN	0	Positive	TP	1	Positive	TP	1
14	48	HR	HSIL (CIN III)	1	Negative	FN	0	Positive	TP	1	Positive	TP	1
15	45	HR	HSIL (CIN III)	1	Negative	FN	0	Positive	TP	1	Positive	TP	1
16	28	HR	HSIL (CIN III)	1	Positive, Focal	TP	1	Positive	TP	1	Positive	TP	1
17	43	HR	HSIL (CIN III)	1	Negative	FN	0	Positive	TP	1	Positive	TP	1
18	33	HR	HSIL (CIN III)	1	Positive, Focal	TP	1	Positive	TP	1	Positive	TP	1
19	42	HR	HSIL (CIN III)	1	Negative	FN	0	Positive	TP	1	Positive	TP	1
20	34	HR	HSIL (CIN III)	1	Negative	FN	0	Positive	TP	1	Positive	TP	1
21	30	HR	HSIL (CIN III)	1	Positive	TP	1	Positive	TP	1	Positive	TP	1
22	36	HR	HSIL (CIN III)	1	Positive	TP	1	Positive	TP	1	Positive	TP	1
23	38	NT	HSIL (CIN III)	1	Positive	TP	1	Positive	TP	1	Positive	TP	1
24	35	NT	HSIL (CIN III)	1	Positive	TP	1	Positive	TP	1	Positive	TP	1
25	28	NT	HSIL (CIN III)	1	Negative	FN	0	Positive	TP	1	Positive	TP	1
26	24	HR	HSIL (CIN III)	1	Negative	FN	0	Positive	TP	1	Positive	TP	1
			AIS, n = 10										
27	36	HR	AIS	1	Negative	FN	0	Positive	TP	1	Positive	TP	1
28	39	16	AIS	1	Positive	TP	1	Positive	TP	1	Positive	TP	1
29	47	NT	AIS	1	Positive	TP	1	Positive, Focal	TP	1	Positive	TP	1
30	30	18	AIS	1	Positive	TP	1	Negative	FN	0	Positive	TP	1
31	36	16	AIS	1	Positive	TP	1	Positive	TP	1	Positive	TP	1
32	31	16	AIS	1	Positive	TP	1	Positive	TP	1	Positive	TP	1
33	31	16	AIS	1	Positive	TP	1	Positive	TP	1	Positive	TP	1
34	31	16	AIS	1	Positive	TP	1	Positive	TP	1	Positive	TP	1
35	36	16	AIS	1	Positive	TP	1	Positive	TP	1	Positive	TP	1
36	43	16	AIS	1	Positive	TP	1	Positive	TP	1	Positive	TP	1
			UIEACa, n = 6										
37	48	NT	UIEACa	1	Positive	TP	1	Positive	TP	1	Positive	TP	1
38	48	NT	UIEACa	1	Positive	TP	1	Positive	TP	1	Positive	TP	1
39	38	NT	UIEACa	1	Positive	TP	1	Positive	TP	1	Positive	TP	1
40	44	16	UIEACa	1	Positive	TP	1	Positive	TP	1	Positive	TP	1
41	41	NT	UIEACa	1	Positive	TP	1	Positive	TP	1	Positive	TP	1
42	37	16+HR	UIEACa	1	Positive	TP	1	Negative	FN	0	Positive	TP	1
			EACaET, n = 8										
43	81	NT	EACaET	1	Positive	FP	1	Negative	TN	0	Positive, P	FP	1
44	72	NT	EACaET	1	Negative	TN	0	Positive	FP	1	Positive, P	FP	1
45	78	NT	EACaET	1	Positive, Focal	FP	1	Positive, Focal	FP	1	Positive, P	FP	1
46	58	NT	EACaET	1	Positive	FP	1	Positive	FP	1	Positive, P	FP	1
47	83	N	EACaET	1	Positive	FP	1	Positive	FP	1	Positive, P	FP	1
48	63	NT	EACaET	1	Positive	FP	1	Positive	FP	1	Positive, P	FP	1
49	72	N	EACaET	1	Positive, Focal	FP	1	Positive	FP	1	Negative	TN	0
50	79	NT	EACaET	1	Positive	FP	1	Positive	FP	1	Positive, P	FP	1

HPV, human papillomavirus status; **N**; negative; **HR**, positive high risk HPV other than types 16 & 18; **NT**, no test results available; **16**, HPV type 16; **18**, HPV type 18; **H&E**, hematoxylin and eosin; **HSP27**, heat shock protein 27; **LSIL**, Low grade squamous intraepithelial lesion; **HSIL**, high grade squamous intraepithelial lesion; **CIN**, cervical intraepithelial neoplasia; **AIS**, adenocarcinoma in situ, endocervix; **UIEACA**, Invasive endocervical adenocarcinoma, usual type; **Perform**, test performance; **TN**, true negative; **TP**, true positive; **FN**, false negative; **Positive, P**, positive, patchy; **FP**, false positive; **0**, negative; **1**, positive
